# Synaptic input changes to spinal cord motoneurons correlate with motor control impairments in a type 1 diabetes mellitus model

**DOI:** 10.1002/brb3.372

**Published:** 2015-09-09

**Authors:** Suzana Ulian Benitez, Everardo Magalhães Carneiro, Alexandre Leite Rodrigues de Oliveira

**Affiliations:** ^1^ Department of Structural and Functional BiologyInstitute of BiologyState University of Campinas13083‐970CampinasSao PauloBrazil

**Keywords:** Diabetes mellitus, motoneuron, nonobese diabetic mice model, spinal cord, synaptic terminals, ventral horn

## Abstract

**Introduction:**

Hyperglycemia is the main cause of diabetic complications, contributing to a widespread degeneration of the nervous system. Nevertheless, the main focus has been the sensory neurons because of neuropathic pain, while the impairments associated with the spinal cord and motor deficits, mostly of those initiated at early stages of the disease, have been poorly investigated. In this way, the present study used the nonobese diabetic mouse model to evaluate the microenvironment around motoneurons at ventral horn of the spinal cord, following prolonged hyperglycemia.

**Methods:**

Adult female mice were divided into two groups: spontaneously diabetic (*n* = 33) and nondiabetic (*n* = 26). Mice were considered hyperglycemic when blood glucose surpassed 400 mg/dL. Following 2 weeks from that stage, part of the animals was euthanized and the lumbar intumescences were obtained and processed for immunohistochemistry and transmission electron microscopy. For immunohistochemistry, the antibodies used for integrated density of pixels quantification were anti‐synaptophysin, anti‐GFAP, and anti‐Iba1. The functional analysis was monitored with the walking track test (CatWalk system) during 4 weeks.

**Results:**

The results revealed significant motor impairment in diabetic animals in comparison to the control group. Such loss of motor control correlated with a significant reduction in presynaptic terminals apposed to the motoneurons. Nevertheless, there were no significant changes in glial reaction in the spinal cord.

**Conclusion:**

Overall, the results herein revealed central nervous system changes at early stages of the disease that may in turn contribute to the motor deficit. Such changes open a new window of investigation in early stages of diabetes to better comprehend motor impairment as a long‐term complication of the disease.

## Introduction

Diabetes mellitus (DM) is the most common metabolic disorder in humans (Beauquis et al. 2008) involving a group of related diseases characterized by hyperglycemia as a result of insufficient insulin secretion, insulin resistance, or both (Gispen and Biessels 2000). The long‐term complications of the disease affect the visual system, kidneys, heart, and blood vessels (Beauquis et al. 2008; Oliveira et al. 2013; Sato et al. 2014). Moreover, the nervous system is also affected leading to complications at the CNS (central nervous system) and PNS (peripheral nervous system) levels (Zochodne et al. 2008). Importantly, both insulin and its receptor are present in the CNS (Gispen and Biessels 2000; Zochodne et al. 2008), and play a modulatory role in synaptic transmission and plasticity (Gispen and Biessels 2000; Northam et al. 2009; Jones 2012). It has been reported that diabetes may affect neurotransmitter pathways (Northam et al. 2009), damaging nerves and affecting cognition (Gispen and Biessels 2000; Jones 2012). Constant hyperglycemia may also affect the blood–brain barrier function (Northam et al. 2009). Sympathetic nervous system is also affected, leading to swollen axons and dendrites in a diabetes model (Schmidt et al. 2003).

It is reported that approximately 50% of diabetic patients develop peripheral neuropathy or damage to the PNS (Zochodne et al. 2008). Such impairments could be only partially prevented with intensive insulin treatment (Gispen and Biessels 2000), which has been reported to increase the risk of dementia following chronic use due to the alterations in metabolic pathways at the CNS (Jones 2012). In more severe cases, at latter stages of diabetes, patients develop polyneuropathy, that is associated with limb numbness, insensitivity to injury, which leads to foot ulceration and amputation, allodynia, and severe intractable pain (Zochodne et al. 2008; Talbot et al. 2010; Francis et al. 2011). Motor incoordination has also been linked to constant falling in type 2 diabetes (Schwartz et al. 2008).

Although much is known about metabolic diabetic complications, the impairments within the CNS, mainly at the spinal cord, affecting motor pathways have been relatively little studied. The primary clinical problems are almost exclusively centered on the neuropathic pain and loss of sensation (Zochodne et al. 2008), lacking a focus on the motor component, which may contribute for motor impairment as a long‐term complication of the disease.

Considering this scenario, the NOD (nonobese diabetic) mouse is a useful tool for studying DM1 (type 1 diabetes mellitus), because it undergoes autoimmune spontaneous *β*‐cell degeneration (Saravia and Homo‐Delarche 2003; Schmidt et al. 2003), with clinical and pathophysiological features suitable to precede translational human studies (Schmidt et al. 2003). In this way, the better understanding of morphological and functional changes, at the ventral horn of the spinal cord, during the initial stages of high hyperglycemia may help the development of better neuroprotective strategies to ameliorate the CNS degeneration during the course of the disease. Due to all of alterations previously described, and considering there is still lack of comprehension of how such impairments are caused and affect motor control, we aimed to investigate glial and synaptic changes at the ventral horn of spinal cord and its correlation to motor alterations during the course of hyperglycemia.

## Materials and Methods

### Animals

Fifty‐nine adult female NOD/Uni mice (4 weeks old) were obtained from the Multidisciplinary Center for Biological Investigation (CEMIB/UNICAMP) and housed under a 12‐h light/dark cycle with free access to food and water. The study was approved by the Institutional Committee for Ethics in Animal Use (CEUA/IB/UNICAMP, prot. no. 1636‐2). The glycemia was monitored twice a week from the fourth week of age. Measurements were carried out by mid‐morning, and the animals were not fasted before the glucose level was measured. Mice were considered hyperglycemic when blood glucose surpassed 400 mg/dL, event that occurred at the age of 21–25 weeks (25 g body weight). Control mice presented glucose levels up to 150 mg/dL. In the present study, mice which developed diabetes were kept alive for two additional weeks, without any treatment (diabetic group) for immunohistochemistry and TEM (transmission electron microscopy). Age‐matched nondiabetic siblings were used as the control group as described previously (Schmidt et al. 2003). For the gait parameter analysis (CatWalk system, Noldus, the Netherlands), siblings and diabetic animals were monitored twice a week for 30 days after the onset of the disease. The number of animals used for each technique is described in Table 1.

**Table 1:** Number of animals used throughout the study, detailed per technical procedure

Technical procedure	Control	Diabetic
Immunohistochemistry synaptophysin	5	9
Immunohistochemistry glial fibrillary acidic protein	9	11
Immunohistochemistry ionized calcium‐binding adapter	5	5
Transmission electron microscopy	4	5
CatWalk	3	3
Total number per group	26	33

### Diabetes and glycemic control

**Figure 1:**
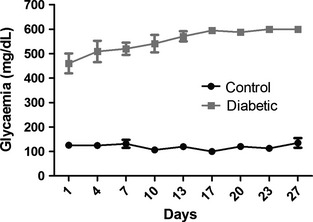
Blood glucose concentration of control (*n* = 7) and diabetic (*n* = 5) animals from the detection of hyperglycemia until euthanasia for morphological evaluation.

The blood glucose concentration samples were obtained from the tail vein and measured by a strip‐operated blood glucose sensor (Accu‐Chek Performa; Roche, São Paulo, Brazil). The glucose concentration in blood serum was in the range of 100–165 mg/dL for control animals, and >400 mg/dL for diabetic animals, at the beginning of the experiment. Fifteen days later, the control animals maintained the glycemia at around 100 mg/dL as previously measured, but the diabetic mice presented increased values (over 600 mg/dL), as reported elsewhere (Schmidt et al. 2003). It was not possible to measure glucose levels above it, as this was the limit of the device scale. Figure 1 shows the blood glucose concentration of animals during the experiments. Diabetic mice demonstrated typical symptoms of diabetes including polydipsia and polyuria.

### Immunohistochemistry for anti‐synaptophysin, anti‐GFAP, and anti‐Iba1

After the predetermined survival period, the animals were anesthetized with a mixture of Kensol (xylasin; 10 mg/kg) and Vetaset (ketamine; 50 mg/kg), and the vascular system was rinsed by transcardial perfusion with PBS (phosphate‐buffered saline, pH 7.4). For the immunohistochemical detection, the animals were fixed by vascular perfusion with 4% formaldehyde in PBS. The lumbar intumescence from each animal was dissected out, post fixed overnight in the same fixative, washed in PBS, and stored in sucrose (20%) for 8 h before freezing. Cryostat transverse sections (12‐*μ*m thick) of the spinal cords were obtained and transferred to gelatin coated slides, dried at room temperature for 30 min, and stored at –21°C until use. The sections were washed three times in PBS and blocked with 3% BSA (bovine serum albumin) in PBS plus 0.2% Tween for 1 h. Primary antibodies are detailed in Table 2. The primary antibodies were diluted in a solution containing 1% BSA in PBS and Tween ×100. All sections were incubated overnight at 4°C in a moist chamber. After washing with PBS, the sections were incubated with the secondary antibody according to the primary host antiserum (1:500; Cyanine Dyes CY‐3, Jackson Immunoresearch, West Groove, PA) for 45 min in a moist chamber at room temperature. The sections were then rinsed in PBS, mounted in a mixture of glycerol/PBS (3:1), and observed in a Nikon Eclipse TS100 inverted microscope (Nikon, Tokyo, Japan). For quantitative measurements, three representative images of each spinal cord were captured at a final magnification of ×200 with a CCD camera (DXM1200i; Nikon) and the software Metamorph 6.3 (Meta Imaging Series; Molecular Devices, Sunnyvale, CA). Immunolabeling pattern of the antibodies used in this work were similar to that observed in other studies (Perez et al. 2013; Spejo et al. 2013). For quantitative measurements, three alternate sections from the same level of the spinal cord (just the right side was chosen for standardization) from each animal (*n* = 5 for diabetic group and *n* = 4 for control group) were used to capture images from the ventral horn at a final magnification of ×200, always using identical settings. Quantification was performed with the enhance contrast and density slicing feature of ImageJ software (version 1.33; National Institutes of Health, Bethesda, MD). In this way, the threshold of each image was manually obtained by comparison with the initially captured images. For synaptophysin immunolabeling, the integrated density of pixels was systematically measured in eight representative areas around each motor neuron dorsolateral lamina IX using a circular sampling area as described previously (Oliveira et al. 2004; Barbizan and Oliveira 2010; De Freria et al. 2012; Freria et al. 2012; Perez et al. 2013; Spejo et al. 2013; for specific review of the method please check supplementary material of Freria et al. 2012). For the analysis of anti‐GFAP and anti‐Iba1 antibodies, the integrated density of pixels was measured in the whole picture in the lateral region of the spinal cord at the ventral horn as reported in a previous work (Perez et al. 2013). The integrated pixel density was calculated for each section and then as a mean value for each spinal cord. The data are represented as mean ± SE (standard error).

**Table 2:** Primary antibodies used for immunohistochemistry

Antibody	Manufacturer/Cat. no.	Anti‐IGG	Mono‐ or polyclonal
Synaptophysin	DakoCytomation/M0776	Mouse	Monoclonal
Glial fibrillary acidic protein	Abcam7779	Rabbit	Polyclonal
Ionized calcium‐binding adaptor protein	Wako/019‐19741	Rabbit	Polyclonal

### Transmission electron microscopy

For TEM, 100 mL of a fixative containing 2.5% glutaraldehyde and 0.5% paraformaldehyde in PBS was perfused through the ascending aorta. The lumbar spinal cords were removed and stored overnight in the same fixative at 4°C. The specimens were then trimmed and osmicated, dehydrated, and embedded in Durcupan ACS (Fluka, Steinheim, Switzerland). Ultrathin sections (60 nm thick) from the L4–L6 segments were collected on formvar‐coated copper grids, and then contrasted with uranyl acetate and lead citrate, and examined under a Leo 906 transmission electron microscope operating at 60 kV.

Neurons with large cell bodies found in the sciatic motoneuron pool of spinal cord were identified as alpha motoneurons by the presence of C‐type nerve terminals (Barbizan and Oliveira 2010; De Freria et al. 2012; Freria et al. 2012). The surface of the cells were then sequentially digitized at a magnification of ×11,300 by using a video camera connected to a computerized system, plus the acquisition feature of the Kontron KS300 software (Zeiss, Jena, Germany). The images were then mounted together using the Adobe Photoshop software (Adobe Systems Software Ireland Ltd., Ireland), and the total perimeter of the neurons was measured. Synaptic terminals apposing the motoneuron somata were identified, and their numbers per 100 *μ*m of cell membrane was calculated by using the measurement tool of the Image Tool software (version 3.0; The University of Texas Health Center at Santo Antonio, TX). The terminals were typed as F (with flattened synaptic vesicles, inhibitory inputs), S (with spherical synaptic vesicles, excitatory inputs), or C (cholinergic inputs) according to the nomenclature of Conradi (1969). In total, two neurons per animal in each group were analyzed (*n* = 5 for diabetic and *n* = 4 for control) according to previous publications (Oliveira et al. 2004; Barbizan and Oliveira 2010; De Freria et al. 2012). The values are shown as a mean ± SE.

### Walking track test

In the walking track test (CatWalk system; Noldus Inc., Wageningen, the Netherlands), the animal crosses a walkway with an illuminated glass floor. A high‐speed video camera Gevicam (GP‐3360; GEViCAM Inc., Milpitas, CA) equipped with a wide‐angle lens (6.0 mm; DF6HA‐1B, Fujinon Corp., China) is positioned underneath the walkway and the paw prints are automatically recorded and classified by the software as the animal moves across the pathway. The measurements were recorded every 3 days for a month, after the detection of diabetes, in the diabetic group. Control group was analyzed in parallel to the hyperglycemic group. Motor function was analyzed for 30 days following hyperglycemia was detected, using the base of support (the average width between the paws), stride length (the distance between successive placements of the paw), and maximum intensity at (%) (the time in seconds since the start of the run that the largest part of the print makes contact with the glass plate, relative to the total duration of contact) of the hind paws parameters of CatWalk system.

### Statistical analysis

The data were analyzed by one‐way (immunohistochemistry and TEM) and two‐way (walking track test) analysis of variance (ANOVA). *P *<* *0.05 (*), *P *<* *0.01 (**), and *P *<* *0.001 (***) were considered statistically significant.

## Results

### DM1 triggers synaptic changes around motoneurons

**Figure 2:**
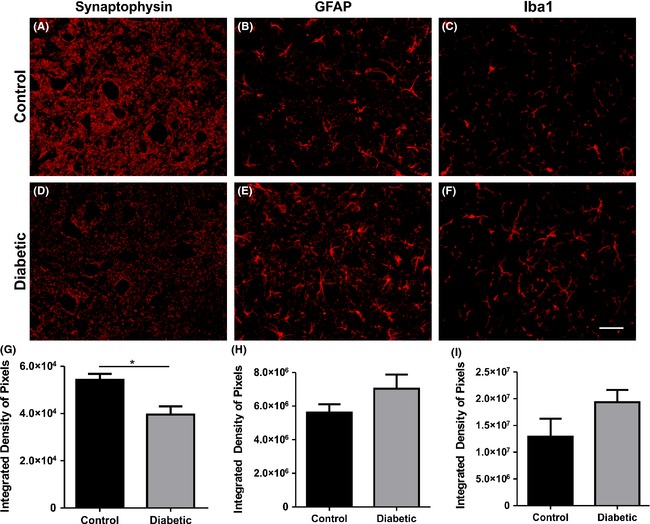
Immunohistochemistry at lamina IX. (A, D) Synaptophysin. (B, E) GFAP (glial fibrillary acidic protein). (C, F) Iba1 (ionized calcium‐binding adapter molecule 1). (G–I) Quantification of the integrated density of pixels of synaptophysin, GFAP, and Iba1, respectively. *P < 0.05.

**Figure 3:**
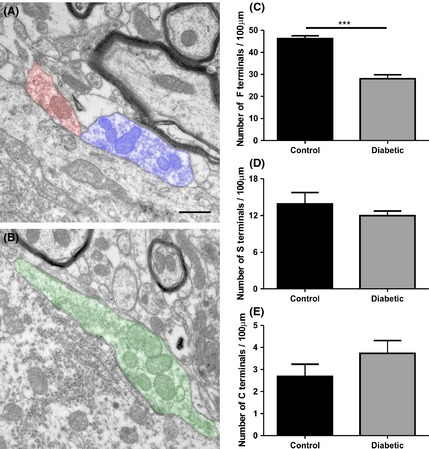
Transmission electron microscopy analysis. (A) Representative image of F terminals (orange) and S terminals (blue) obtained from a control specimen. (B) Representative image of C terminals (green) obtained from a control specimen. (C–E) Graphs of the number of F, S, and C terminals per 100 *μ*m of cell membrane of the motoneuron. ***P < 0.001.

Significant synaptic density decrease was detected in the diabetic mice as seen by anti‐synaptophysin labeling (*P* < 0.05) (Fig. 2A, D, and G, control 54,210 ± 2490 *n* = 5 and diabetic 39,540 ± 3475 *n* = 9). Such finding is consistent with the TEM evaluation that allows a fine observation of the surface of the spinal motoneurons. In this sense, a decrease in F terminal covering in apposition to the motoneuron somata (Fig. 3C, control – 46.18 ± 1.35%, *n* = 4; diabetic – 28.08 ± 1.76%, *n* = 5; *P* < 0.001) was found, indicating a significant reduction of putative inhibitory inputs to alpha motoneurons. On the contrary, no statistical change in S terminal apposition was depicted (Fig. 3D, control – 13.88 ± 1.86%, *n* = 4; diabetic – 11.96 ± 0.755%, *n* = 5), suggesting a possible overall relative increase in excitation that may trigger excitotoxic events. No significant changes in cholinergic C terminals (Fig. 3E, control – 2.688 ± 0.552%, *n* = 4; diabetic – 3.732 ± 0.581%, *n* = 5) could be detected during hyperglycemia.

### Two weeks of hyperglycemia do not cause significant changes in glial reaction

There was no significant increase in GFAP immunolabeling in the diabetic mice as compared to the control group (Fig. 2B, E, and H; control – 1.29 × 10^7^ ± 3.34 × 10^6^, *n* = 9; diabetic – 1.93 × 10^7^ ± 2.31 × 10^6^, *n* = 11; integrated density of pixels measured per picture area). The same pattern was depicted for Iba1 immunostaining (Fig. 2C, F, and I; control – 5.61 × 10^6^ ± 4.98 × 10^5^, *n* = 5; diabetic – 7.04 × 10^6^ ± 8.38 × 10^5^, *n* = 5; integrated density of pixels measured per picture area).

### Diabetic mice develop motor impairments at the initial stage of the disease

**Figure 4:**
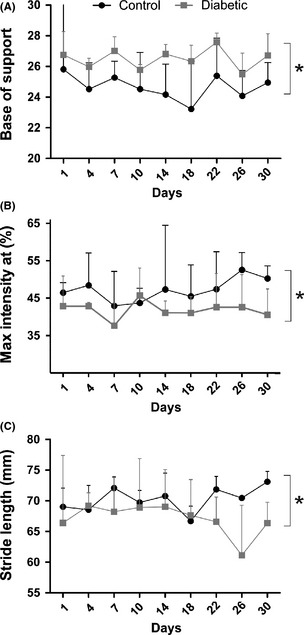
Walking track test carried out using the CatWalk system (Noldus Inc., Holland). (A) Base of support. (B) Maximum intensity at (%). (C) Stride length (mm). *P < 0.05.

In order to access the effects of prolonged hyperglycemia on the locomotor behavior we used the walking track test. The evaluation was performed on the CatWalk system, which provides accurate measurements of paw prints as well as pressure during spontaneous locomotion. The results revealed that maximum intensity at (%) and base of support parameters were increased (*P* = 0.0161 and *P* = 0.0451, respectively; Fig. 4A and B), while stride length was decreased in diabetic mice (*P* = 0.0424) (Fig. 4C). Such results are consistent with the immunohistochemical findings regarding synaptic stripping; indicating that hyperglycemia quickly affects motor skills, possibly by harming spinal descending pathways as well as local spinal cord networks.

## Discussion

The present work reports important changes at the spinal cord microenvironment during the initial course of DM1 and its correlation with motor coordination deficits, indicating that synaptic changes occur soon after hyperglycemia is installed in diabetic individuals. The impact of DM on CNS has already been related to learning and memory problems. Such deficiencies are associated to vascular dementia as well as Alzheimer's disease. Also, impairments in mental and motor responsiveness have been proposed (Gispen and Biessels 2000), although how such complications happen into the spinal cord are still poorly understood.

Diabetes mellitus leads to damage of neurons, and hampering regenerative capacity (Zochodne et al. 2008). In this sense, a previous work reported that sensory neurons are first affected, resulting in loss of skin innervation (Zochodne et al. 2008). Later, sensory neurons undergo atrophy and eventual degeneration. Contrarily to that, although motoneurons are preserved, a loss of neuromuscular junctions takes place during the course of the disease (Zochodne et al. 2008; Francis et al. 2011). It is also reported that sympathetic neuritic dystrophy occurs within 3–5 weeks after the onset of hyperglycemia (Schmidt et al. 2003).

Most studies relating hyperglycemia and CNS degeneration focused on neuronal and glial cells at the dorsal horn of the spinal cord (Wang et al. 2007; Talbot et al. 2010; Chen et al. 2011; Crown 2012; Dauch et al. 2012). On the contrary, there is a lack of publications aiming at understanding changes in the ventral horn. Thus, motoneurons have not been widely considered among the primary targets of DM and, although they have been suspected to be less susceptible to diabetic polyneuropathy, this has not been validated in any experimental system (Zochodne et al. 2008). In this respect, the present work shows considerable changes within motoneuron microenvironment after 2 weeks of hyperglycemia.

Spinal cord is a complex part of the CNS containing glial cells and neurons. Such neurons convey supraspinal inputs to the periphery as well as transmit afferent inputs, thus coordinating the flow of information regarding sensation and limb movement. It is known that the spinal dorsal horn is a critical site for the transmission and modulation of nociception, receiving glutamatergic inputs from primary afferents and local interneurons (Chen et al. 2011). In this sense, it is known that an increase in glutamatergic inputs into the dorsal horn of the spinal cord occur in diabetic rats (Wang et al. 2007; Chen et al. 2011). Therefore, there is an imbalance between excitatory and inhibitory inputs at the dorsal horn, which might contribute to neuropathic pain. It also may trigger an imbalance of inputs in other areas of the spinal cord as well, such as the ventral horn. It is important to emphasize that most of the glutamatergic input to the motoneurons arises from dorsal root ganglia sensory neurons, which are vesicular glutamate transporter 1 positive. In a previous study, the section of dorsal roots was able to deplete near to all glutamatergic synapses in the ventral horn (Oliveira et al. 2003). In this way, alterations of DRG neurons by hyperglycemia may directly affect spinal motoneuron inputs, generating the abovementioned input imbalance.

Based on the consequences mentioned above, associated with metabolic complications of the diabetes, such as motor and sensory nerve conduction deficits (Obrosova et al. 2005), it is feasible to suggest that hyperglycemia also affects the ventral horn microenvironment. Such changes include significant loss of inhibitory terminals in apposition to motoneuron cell bodies, as seen by synaptophysin immunolabeling and TEM. Our work is consistent with previous data, in which reduction in synaptophysin immunolabeling was depicted 20 weeks after the onset of the disease (Jiang et al. 2010). We suggest that such changes occur much sooner than expected. Such immediate changes may contribute to generating an imbalance between excitatory and inhibitory inputs, contributing to motor control impairment and gait alterations, as seen herein.

Additionally to loss of synapses, the close relation between astrocytes and the blood–brain barrier makes them early sensors for variations in glucose homeostasis. It has been already shown activation of such cells in the hippocampus (Beauquis et al. 2008) and at the spinal cord in a type 2 DM model (Liao et al. 2011; Dauch et al. 2012; Ren et al. 2012). On the other hand, it has also been reported a reduction in GFAP immunolabeling at the spinal cord following the course of the disease (Afsari et al. 2008). Our results showed no statistical upregulation of GFAP, although there is a trend of increasing. Also, microglial activation was present in the diabetic material, although no statistical differences in comparison to the control mice could be obtained at such early stage of the disease. It has already been shown that microglia activation occurs at the dorsal horn of spinal cord at early stages of diabetes (Talbot et al. 2010) and it is widely related with the development of neuropathic pain (Wodarski et al. 2009; Talbot et al. 2010; Wang et al. 2014). Although we were unable to find statistical increase of glial reaction at such early stage of the disease, it is possible that the trend of gliosis become significant at later survival times, even in the ventral horn. This may be investigated in future follow‐up studies.

Despite no significant changes in glial cells, the presence of early changes in the synaptic terminals around motoneurons, following 2 weeks of hyperglycemia, is of great relevance, since motoneurons constitute a nucleus of integration of a variety of sensory and motor information toward and from the PNS. Motoneurons play a critical role in coordinating muscle fibers for accurate movement. In humans, the presence of high glucose plasma levels lead to peripheral neuropathy at later stages, which is associated with a decline in the motor compound action potential, unstable neuromuscular junction, and abnormal foot positioning, associated with altered distribution of plantar pressure during standing or walking (Zochodne et al. 2008). Nevertheless, patients do not report progressive and widespread muscle weakness as observed in motoneuron diseases, such as ALS (Zochodne et al. 2008). Locomotor impairments could be the summation of different factors such as oxidative stress (Obrosova et al. 2005; Zochodne et al. 2008), disturbances in axonal conduction (Gispen and Biessels 2000), slowing of axonal conduction velocity (Gispen and Biessels 2000; Obrosova et al. 2005), vascular dysfunction, reduced nerve blood flow (Gispen and Biessels 2000; Zochodne et al. 2008), and alterations in trophic support which might contribute to metabolic changes in the motoneurons. It is also reported that the responsiveness to acute pain is reduced in NOD diabetic mice (Obrosova et al. 2005). Altogether such factors may contribute to the imbalance between excitatory and inhibitory inputs at the motoneuron cell body, as shown in the present work, contributing to the significantly decreased performance in comparison to the control counterpart.

An additional novelty of the present work is the use of the CatWalk system as an automatized gait acquisition apparatus for diabetes studies. Such system has been widely used to report functional impairments due to peripheral nerve lesion, spinal cord lesion, root transection, and neurodegenerative diseases. Nevertheless, the evaluation of motor disturbances as a result of DM1 has not been reported previously with this approach. The maximum contact at (%) parameter is reported as increased and the base of support parameter is also affected after spinal cord injury (Hamers et al. 2001), and it is also altered in diabetic mice, indicating important changes in the motor circuitry. Overall, the walking track test provided accurate motor control and coordination analysis during the course of early diabetes, and the results were compatible to previous studies investigating spinal cord injury, neurodegenerative diseases, peripheral nerve injury, and corticospinal tract damage (Starkey et al. 2005; Deumens et al. 2007; Chuang et al. 2010; Figley et al. 2014). Nevertheless, evaluation of greater number of subjects at different stages of the disease as well as additional gait parameters may provide further details and reinforce the findings described herein.

## Conclusion

The present study brings new early effects of DM1 within the spinal cord at the ventral horn and its correlation with motor impairments. Such findings reinforce the possible correlation between synaptic changes and motor complications, providing a new perspective regarding presynaptic input retraction from motoneuron surface as the result of early installed hyperglycemia.

## Conflict of Interest

None declared.
